# Suicide prevention and depression apps’ suicide risk assessment and management: a systematic assessment of adherence to clinical guidelines

**DOI:** 10.1186/s12916-019-1461-z

**Published:** 2019-12-19

**Authors:** Laura Martinengo, Louise Van Galen, Elaine Lum, Martin Kowalski, Mythily Subramaniam, Josip Car

**Affiliations:** 1 Centre for Population Health Sciences, Lee Kong Chian School of Medicine, Nanyang Technological University, 11 Mandalay Road, Level 18, Singapore, 308232 Singapore; 2 Section of Acute Medicine, Department of Internal Medicine, Amsterdam UMC Location VUmc, Amsterdam, Netherlands; 3 School of Clinical Sciences, Faculty of Health, Queensland University of Technology, Brisbane, Australia; 4 University of Split, School of Medicine, Split, Croatia; 5 Research Division, Institute of Mental Health, Singapore, Singapore; 6 Neuroscience & Mental Health Research Programme, Lee Kong Chian School of Medicine, Nanyang Technological University, Singapore, Singapore; 7 Global Digital Health Unit, Department of Primary Care and Public Health, School of Public Health, Imperial College London, London, UK

**Keywords:** Suicide, Suicide prevention, Depression, Mobile applications, Apps, Telemedicine, mHealth, Crisis intervention, Crisis helpline

## Abstract

**Background:**

There are an estimated 800,000 suicides per year globally, and approximately 16,000,000 suicide attempts. Mobile apps may help address the unmet needs of people at risk. We assessed adherence of suicide prevention advice in depression management and suicide prevention apps to six evidence-based clinical guideline recommendations: mood and suicidal thought tracking, safety plan development, recommendation of activities to deter suicidal thoughts, information and education, access to support networks, and access to emergency counseling.

**Methods:**

A systematic assessment of depression and suicide prevention apps available in Google Play and Apple’s App Store was conducted. Apps were identified by searching 42matters in January 2019 for apps launched or updated since January 2017 using the terms “depression,” “depressed,” “depress,” “mood disorders,” “suicide,” and “self-harm.” General characteristics of apps, adherence with six suicide prevention strategies identified in evidence-based clinical guidelines using a 50-question checklist developed by the study team, and trustworthiness of the app based on HONcode principles were appraised and reported as a narrative review, using descriptive statistics.

**Results:**

The initial search yielded 2690 potentially relevant apps. Sixty-nine apps met inclusion criteria and were systematically assessed. There were 20 depression management apps (29%), 3 (4%) depression management and suicide prevention apps, and 46 (67%) suicide prevention apps. Eight (12%) depression management apps were chatbots. Only 5/69 apps (7%) incorporated all six suicide prevention strategies. Six apps (6/69, 9%), including two apps available in both app stores and downloaded more than one million times each, provided an erroneous crisis helpline number. Most apps included emergency contact information (65/69 apps, 94%) and direct access to a crisis helpline through the app (46/69 apps, 67%).

**Conclusions:**

Non-existent or inaccurate suicide crisis helpline phone numbers were provided by mental health apps downloaded more than 2 million times. Only five out of 69 depression and suicide prevention apps offered all six evidence-based suicide prevention strategies. This demonstrates a failure of Apple and Google app stores, and the health app industry in self-governance, and quality and safety assurance. Governance levels should be stratified by the risks and benefits to users of the app, such as when suicide prevention advice is provided.

## Introduction

In 2016, there were an estimated 800,000 suicides globally, and approximately 16,000,000 suicide attempts [1]. Despite reports of almost 33% decrease in the global age-standardized mortality rate from suicide between 1990 and 2016 [2, 3], suicide remains one of the leading causes of preventable deaths in both developing and developed countries. Sixty percent of individuals with suicidal ideation transitioned to a first attempt within a year of onset [4], a significant figure considering that half to two thirds of suicide deaths occur in the first attempt [5, 6]. The risk of suicide increases with access to means of suicide, personal or family history of mental health disorders and suicide attempts, and psychiatric comorbidity. Over 90% of people who died from suicide were affected by depression, alcohol abuse, or both [5, 7]. Suicide prevention programs targeting one or more of these factors successfully decrease the number of suicides [8, 9]. An important and widespread component of suicide prevention strategies are crisis helplines, which provide timely and anonymous advice to callers at current risk of suicide and are effective in deterring active suicidal thoughts [10–12].

Timely identification of persons at risk of suicide is critical to ensure adequate provision of care. Family physicians (FPs) play an important role as most individuals who died by suicide visited their FP in the month preceding death [5], and about 90% consulted their FP several times the prior year [13]. Nevertheless, efforts by healthcare providers to identify patients at risk face significant hurdles, particularly the unwillingness of affected individuals to disclose suicidality fearing loss of autonomy, overreaction, and stigma [14, 15].

Forty percent of people with suicidal thoughts or behaviors do not seek medical care [16], or may not have access to healthcare, particularly in developing countries. Digital interventions delivered online or through mobile devices may increase access to help and mental health care. Patients feel more at ease discussing mental health conditions online than in a face-to-face encounter [17], and consider the Internet accessible, affordable, and convenient [18].

Over the last decade, the health app market has grown to include about 318,000 apps [19], of which more than 10,000 are mental health apps [20], making selection of an appropriate app cumbersome, particularly for lay users [21]. Digital mental health interventions seem to offer a promising alternative to face-to-face visits [22, 23]. However, very few apps available in app stores have been evaluated in clinical trials [24–28] or by regulatory bodies like the FDA [29].

Previous research on the use of digital health for suicide prevention focused on highlighting features of an ideal intervention [30], systematically reviewing the effectiveness of online interventions and mobile apps [31, 32], app store descriptions of apps [33], or assessing suicide prevention strategies offered by apps [34]. However, none of these studies evaluated suicide prevention advice offered by apps. Given this and the high turnover of apps [35], we conducted a comprehensive assessment of suicide prevention apps available on Google Play and Apple’s App Store worldwide, as well as assessing the suicide prevention advice offered by depression management apps.

## Methods

The aims of this study were:

1. To systematically assess depression and suicide prevention apps’ adherence to evidence-based clinical guidelines on:
  - Strategies for suicide prevention;
  - Type and quality of advice given when the user is at risk of attempting suicide; and
2. To analyze the response of chatbot apps to a user who appears to be at risk of attempting suicide using simulated patient scenarios.

Systematic review methodology was adapted for the app search, selection, assessment, and data analysis.

### App selection

A systematic search on Apple’s App Store and Google Play using the 42matters (https://42matters.com/) was performed in January 2019 using the terms “depression,” “depressive,” “depress,” “mood disorders,” “suicide,” and “self-harm.” The search was limited to medical, lifestyle, health and fitness, and education categories, with no country restrictions. The search engine retrieved the name, category, developer, app store description, date of first release and current version, ratings and number of raters (for iOS only), link to website, and market URL for each app.

### Inclusion criteria

- App targets people suffering from depression; or
- Assesses suicide risk; or
- Provides advice to prevent users from attempting suicide; or
- Follows a “call to action” model. We defined “call to action” as a message delivered by the app using active language and addressed to the user inviting her/him to take action to prevent the urge of hurting her/himself, for example “If you feel suicidal, please call the following number…”; or
- Provides a link for the user to activate a phone call to a crisis helpline directly through the app
- AND
- App has been uploaded or updated from 1st January 2017 onwards
- App is free or requires payment to download/use and is available in Apple’s App Store or Google Play
- App is in English

### Exclusion criteria

- App is aimed at healthcare providers (physicians, psychologists, counselors, others), or the support network of the person at risk of suicide or community gatekeepers
- Offers teleconsultation services with physicians, psychologists, counselors, or other healthcare providers
- App content is not interactive (e.g., books, music playlists, wallpapers, others), does not ask the user to act, or does not provide a direct link to a crisis helpline through the app
- App refers to self-harm with non-suicidal intent
- App consists of a standalone depression screening questionnaire
- App was removed from the app stores at the time of download, required a sign-up code provided by an institution, or could not be used after two attempts due to technical problems

**Fig. 1:**
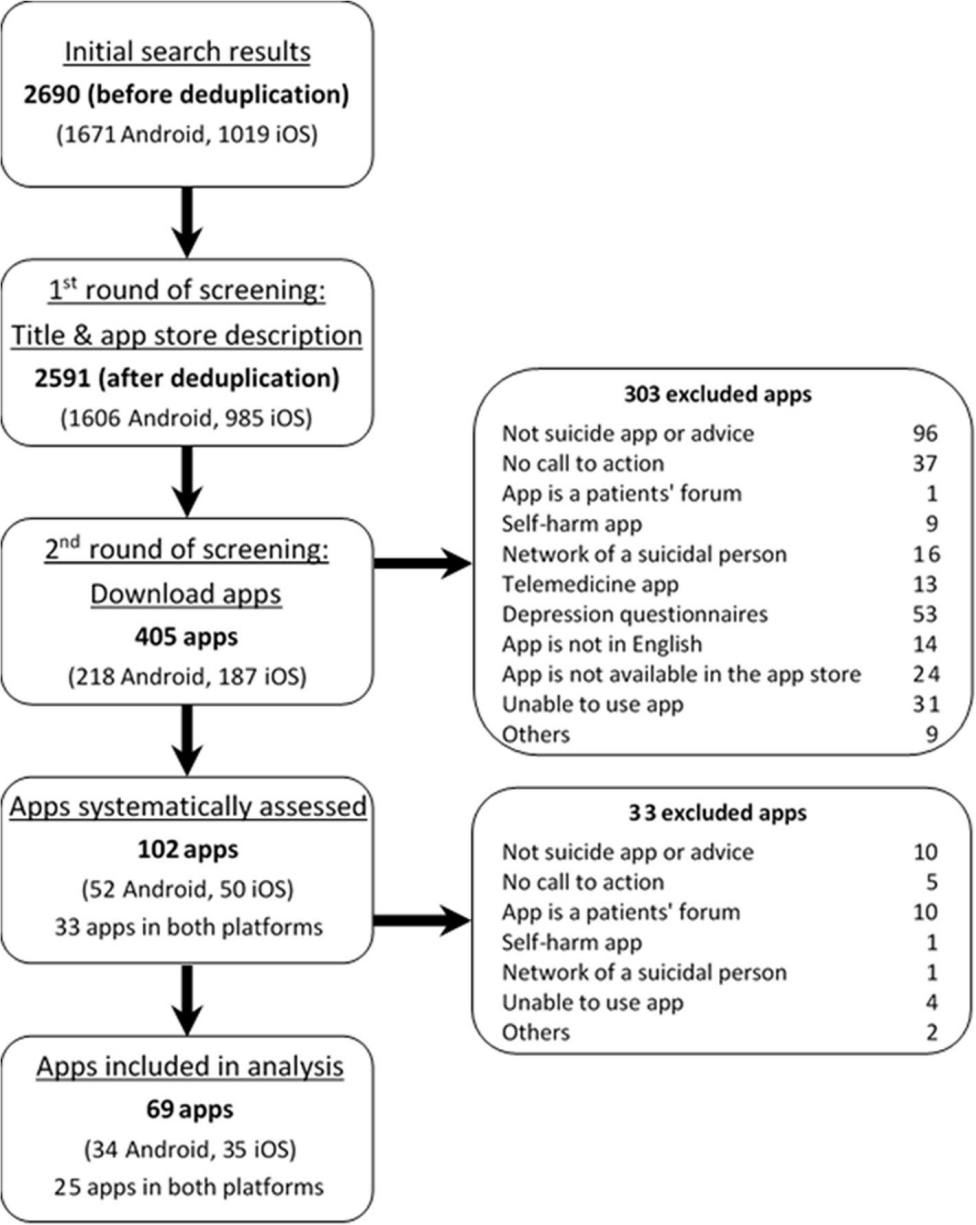
App selection flowchart

The app selection process is presented as a flowchart [36] (Fig. 1).

### Development of the assessment criteria

The assessment criteria were developed by the research team and comprised three main components (Additional file 2: Table S2):

- *General attributes of the app*, including cost and ratings, target user groups, data security measures adopted to ensure user’s privacy, app crashes or malfunction, and who developed the app.
- *Strategies offered by the app to prevent or manage suicidality in a person at risk,* based on evidence-based clinical guidelines (as a prerequisite for their potential of effectiveness) from the UK [37, 38], USA [11, 39], and WHO [40]. The criteria comprised 50 questions organized in six domains:

1. *Tracking of mood and suicidal thoughts*, to assess acute risk of suicide, including users’ mood, triggers for suicidal thoughts, suicide plans and protective factors (reasons for living, plans for the future, coping or problem-solving skills)
2. *Development of a safety plan*, defined as a structured, six-step, standardized list of strategies and contact details of members of his/her support network that a person at risk of suicide can use during a crisis [41].
3. *Recommendation of activities* to deter suicidal thoughts, and follow-up on outcomes.
4. *Information and education*, educational articles on signs of suicidality, risk factors and triggers of suicide, and safety planning. Information included lists of crisis helpline numbers or emergency contact information
5. *Access to support networks*, including saving the contact information of people from the user’s support network (family, friends, and primary healthcare provider) and ability to share information with them
6. *Access to emergency counseling* provided by a healthcare professional or a crisis helpline the user can contact directly through the app, or through a chatbot, e.g.,

- *Trustworthiness of information* provided by the app was adapted from the Health on the Net Foundation Code of Conduct (HONcode) [42] that evaluates the reliability of information based on citations, justification of claims, and authority of information, as well as for adherence to ethical standards of transparency, privacy, and advertising policies.

### App assessment

We followed a systematic, two-step process to select apps for inclusion. First, two investigators (LM and MK) screened app store descriptions of all retrieved apps in parallel. Included apps were then downloaded and screened again according to predefined inclusion and exclusion criteria. Any uncertainty regarding the inclusion or exclusion of apps was resolved by discussion between the assessors. About 20% of apps were assessed by both researchers (LM and MK) to ensure consistent application of assessment criteria, after which the remaining apps were assessed by either one of the researchers. Interrater reliability for apps assessed in parallel by both assessors was calculated using Cohen’s kappa (*κ*). The assessment was considered reliable if the interrater agreement was equal to or higher than 0.6 (substantial or almost perfect agreement) [43].

Apps were assessed using an iPhone 5c (iOS 10.3.3) or iPhone 7 (iOS 11.4.1) and a Sony XPERIA XZs (Android 8.0.0). For apps available on both platforms, both versions were assessed to account for potential differences in app functionalities, and counted as an individual app. We assessed paid and free apps without add-ons available as in-app purchases.

To further ensure consistency in the assessment process, we created a user persona which included demographics, medical diagnosis, potential answers to self-reported questionnaires, and opening statements to converse with a chatbot-based app.

### Data analysis

Descriptive statistics were used to analyze the data. To compare the functionalities and trustworthiness of depression management and suicide prevention apps, a significance test for categorical variables was used: chi-square test if each category contained more than ten variables and two-tailed Fisher’s exact test if any of the categories in the contingency table were below ten. Statistical significance was set at *p* <  0.05.

## Results

### App search

The search strategy retrieved 2591 apps (1606 Android and 985 iOS) after duplicates were removed. Screening yielded 102 apps, of which 69 apps met inclusion criteria and were systematically assessed (9 Android apps, 10 iOS apps, and 25 apps available on both platforms). Twenty-three percent (23/102) of apps were assessed by both assessors with substantial agreement (*κ* = 0.730 (95% CI, .700 to .759), *p* < .0005). Figure 1 summarizes the app search and selection process. Additional file 1: Table S1 lists all the assessed apps and the suicide prevention strategies offered by each app.

### General attributes of apps

The analysis included 20/69 (29%) depression management apps, 46/69 (67%) suicide prevention apps, and 3/69 (4%) apps offering depression management and suicide prevention. Table 1 provides a summary of app attributes. A total of 7/20 (35%) Android depression management apps, including three chatbots, were downloaded > 50,000 times, of which three apps (Moodpath [44], Wysa [45], and Youper [46]) were downloaded > 500,000 times and two apps (7 Cups [47] and Pacifica [48]) were downloaded > 1,000,000 times. An iOS version was available for these apps. Conversely, the number of downloads for suicide prevention apps ranged from 5 to > 10,000.

**Table 1:** General attributes of apps

Feature	DM (*n* = 20)	DM and SP (*n* = 3)	SP (*n* = 46)	Total (*n* = 69)	p*
Cost of the app					
Free	8 (40%)	1 (33%)	44 (98%)	53 (77%)	*< 0.001*
In-app purchases	10 (50%)	2 (67%)	2 (2%)	14 (20%)	
Paid	2 (10%)	–	–	2 (3%)	
App category on the app store					
Education	–	–	18 (39%)	18 (26%)	*< 0.001*
Health & Fitness	15 (75%)	–	20 (44%)	35 (51%)	
Lifestyle	1 (5%)	3 (100%)	–	4 (6%)	
Medical	4 (20%)	–	8 (17%)	12 (17%)	
App ratings					
Not enough ratings	–	2 (67%)	18 (39%)	20 (29%)	*0.001*
1★–3.5★	2 (10%)	–	5(11%)	7 (10%)	
3.5★–5★	18 (90%)	1 (33%)	23 (50%)	42 (61%)	
Target group					
General population	20 (100%)	3 (100%)	34 (74%)	57 (83%)	0.137
Students	–	–	9 (20%)	9 (13%)	
Veterans	–	–	3 (6%)	3 (4%)	
Number of suicide prevention strategies					
1–2	3 (15%)	2 (67%)	11 (24%)	16 (23%)	0.374
3	10 (50%)	–	14 (30%)	24 (35%)	
4	5 (25%)	–	12 (26%)	17 (25%)	
5	1 (5%)	1 (33%)	5(11%)	7 (10%)	
6	1 (5%)	–	4 (9%)	5 (7%)	
Directly connect to emergency helplines					
No	11 (55%)	1 (33%)	10 (22%)	22 (32%)	*0.019*
Yes	9 (45%)	2 (67%)	36 (78%)	47 (68%)	
User can remain anonymous					
No	7 (35%)	1 (33%)	3 (7%)	11 (16%)	*0.009*
Yes	13 (65%)	2 (67%)	43 (93%)	58 (84%)	
Password-protected account					
No	8 (40%)	1 (33%)	37 (80%)	46 (67%)	*0.002*
Yes	12 (60%)	2 (67%)	9 (20%)	23 (33%)	
App crashes or malfunctions					
No	17 (85%)	3 (100%)	37 (80%)	57 (83%)	0.857
Yes	3 (15%)	–	9 (20%)	12 (17%)	
App was created or commissioned by					
Government body, university	2 (10%)	–	19 (41%)	21 (30%)	*0.02*
NGO, healthcare providers	10 (50%)	3 (100%)	18 (39%)	31 (45%)	
Private developer	8 (40%)	–	9 (20%)	17 (25%)	
Export data (pdf/excel/other)					
No	12 (60%)	3 (100%)	42 (91%)	57 (83%)	*0.012*
Yes	8 (40%)	–	4 (9%)	12 (17%)	

*DM* depression management*, SP* suicide prevention; *In italics, statistically significant *p* values (< 0.05)

Most apps were free to download and use. Ten (50%) depression management apps and two (67%) depression management and suicide prevention apps offered in-app purchases for cognitive behavioral therapy-based programs, access to a health provider, or a workplace wellness program. One suicide prevention app included in-app payment for voluntary donations. Forty-five percent of all apps were created or commissioned by healthcare providers or non-profit organizations. In addition, 19/46 (41%) suicide prevention apps were created or commissioned by a government organization or university.

Fifty-one percent of all apps were categorized as “Health and Fitness” in their app store descriptions, while less than 20% of apps were categorized as “Medical.” Educational apps accounted for 18/46 (39%) suicide prevention apps.

### Strategies to manage a person at risk of suicide

Most apps included at least three suicide prevention strategies (see Tables 1 and 2), more commonly emergency contact information (65/69 apps, 94%), direct access to a crisis helpline (46/69 apps, 67%), and suicide-related education (35/69 apps, 51%). A total of 5/69 apps (7%) offered all six strategies. Table 3 presents examples of apps complying with all suicide prevention strategies. Additional file 1: Table S1 provides a detailed description of the strategies used by each app. A description of findings for each strategy is provided below.

**Table 2:** Strategies offered by the apps

Type of strategies	DM (*n* = 20)	DM and SP (*n* = 3)	SP (*n* = 46)	Total (*n* = 69)
Tracking of mood or suicidal thoughts	17 (85%)	1 (33%)	10 (22%)	28 (41%)
Safety plan development	2 (10%)	–	24 (52%)	26 (38%)
Offer activities to deter suicidal thoughts	15 (75%)	1 (33%)	17 (37%)	33 (48%)
Information and education				
Suicide-related	2 (10%)	3 (100%)	30 (65%)	35 (51%)
Emergency contact information	20 (100%)	3 (100%)	42 (91%)	65 (94%)
Access to support networks	2 (10%)	1 (33%)	25 (54%)	28 (41%)
In-app access to emergency counseling				
By counselor	–	–	9 (20%)	9 (13%)
By emergency helpline	9 (45%)	1 (33%)	36 (78%)	46 (67%)

*DM* depression management, *SP* suicide prevention

**Table 3:** Examples of apps offering all six suicide prevention strategies

Stay Alive [49] is an app developed by a UK-based non-government-organization (NGO) (Grassroots Suicide Prevention) that provide users a comprehensive, customizable safety plan template that includes adding contact data for key members of the user’s support network, suicide-related information, grounding and relaxation exercises and direct access to emergency helplines through the app.	

ReMinder App [50] is an app developed by an Australia-based NGO (On the Line) that offers users a customizable template to develop their safety plan using a combination of free text and pre-added options for users to choose from. This app assess the user’s mood using a self-reported depression test (K-10), allows users to save multimedia files to use when in crisis, provides information through Tweeter feed and access to emergency helplines and members of the user’s support network through the app.	

#### Tracking of mood and suicidal thoughts

Seventeen depression management apps (17/20, 85%), 1/3 (33%) depression management and suicide prevention app, and 10/46 (22%) suicide prevention apps tracked users’ mood or suicidal behavior. Depression management apps assessed users’ mood using self-developed questions or a validated questionnaire (Patient Health Questionnaire-9 (PHQ-9) [50]), while eight chatbot apps also assessed users’ suicidal behavior. Conversely, five suicide prevention apps assessed users’ mood and seven assessed users’ suicidal thoughts or behaviors. None of the apps enquired about risk factors, triggers, or protective factors, and only one checked past history of suicide.

#### Safety plan development

Only 2/20 (10%) depression management apps and 24/46 (52%) suicide prevention apps offered users a template to develop a safety plan, and all but one included guidance to complete the safety plan. Only 11 apps included all safety plan steps as developed by Stanley and Brown [41]. The most common missing steps were a list of activities to deter suicidal thoughts and access to users’ support network. In seven apps, the safety plan was one component in a more comprehensive suicide prevention strategy that included educational articles, mood and suicidality assessment, and access to support network and crisis helplines. Only four apps allowed the user to share the safety plan with a member of his/her support network.

#### Recommendation of activities to deter suicidal thoughts

Fifteen depression management apps (15/20, 75%), 1/3 (33%) depression management and suicide prevention app, and 17/46 (37%) suicide prevention apps offered activities aimed to enhance wellbeing, improve mood, or discourage suicidal thoughts, including mindfulness, or another meditation technique, hobbies or outdoor activities, exercise, and healthy lifestyle advice.

#### Information and education

Two depression management apps (2/20, 10%), 3/3 (100%) depression management and suicide prevention apps, and 30/46 (65%) suicide prevention apps provided information on suicide signs, triggers, risk factors and prevention strategies, and how to complete a safety plan. Furthermore, all except two suicide prevention apps providing access to users’ support network and available on both platforms (*n* = 4, 9%) provided emergency contact information including crisis helpline telephone numbers, messaging service numbers, or links to relevant websites. The information in 49 apps was specific for one or several countries, limiting its global usability.

#### Access to support networks

Two depression management apps (2/20, 10%), 1/3 (33%) depression management and suicide prevention apps, and 25/46 (54%) suicide prevention apps allowed users to store members of their support network’s contact details. In 15 apps, including two depression management apps and 13 suicide prevention apps, this functionality was included in a safety plan.

Fourteen suicide prevention apps (14/46, 30%) allowed users to contact members of their support network directly from the app. A subset of these apps (8/46 apps, 17%) used a simple interface aimed exclusively at facilitating immediate, often simultaneous communication with one or several support network members via a telephone call or text message.

#### Access to emergency counseling

Nine suicide prevention apps (9/46, 20%) provided emergency access to trained counselors directly through the app. All but one app were developed by public institutions or non-governmental organizations (NGOs). Three apps specifically targeted veterans and three apps, university students.

In total, 11/20 (24%) depression management apps, 1/3 (33%) depression management and suicide prevention app, and 36/46 (78%) suicide prevention apps offered direct contact to a crisis helpline through the app. The accuracy and functionality of crisis helpline numbers provided by the apps was verified by performing an online search and found to be faulty in six (9%), four depression management, and two suicide prevention apps (Table 4).

**Table 4:** Inaccurate crisis helplines

An important feature often found in depression management and suicide prevention apps is the inclusion of a crisis helpline telephone number that would ideally activate a telephone call directly through the app. As part of our assessment, we checked the accuracy and functionality of the telephone numbers provided by the apps. Six apps, (two apps available in Android and iOS, and two Android apps) provided crisis helpline telephone numbers that were either non-existent (dummy number), non-functional (dialed number failed to connect users to the helpline), or the number provided was linked to an organization offering non-evidence-based treatments. Two of these apps, available in both app stores, had been downloaded more than one million times each. We informed app developers of our findings and two popular apps have since rectified the errors. Providing an uncontactable phone number, particularly to people going through an emergency, potentially risks the lives of highly vulnerable people and constitutes a severe breach of ethical standards.	

### HONcode principles

In general, there were marked variations regarding compliance with HONcode principles (Table 5). Most apps included a privacy policy in their app store description or within the app (44/69, 64%) and provided an accurate email address for users to contact the developers (66/69, 96%), and all apps were advertisement-free, although one suicide prevention app with Android and iOS versions asked for voluntary donations to maintain the app.

**Table 5:** Number of apps in each category complying with HONcode principles

HONcode principles	DM (*n* = 20)	DM and SP (*n* = 3)	SP (*n* = 46)	Total (*n* = 69)	p*
*Authoritative*: qualifications of the authors are indicated	8 (40%)	2 (67%)	3 (7%)	13 (19%)	*< 0.001*
*Complementarity*: information should support, not replace, the doctor-patient relationship	9 (45%)	2 (67%)	19 (41%)	30 (43%)	0.688
*Privacy*: respect the privacy and confidentiality of personal data submitted by the user	14 (70%)	3 (100%)	27 (59%)	44 (64%)	0.327
*Attribution*: cite the source(s) of published information, date medical, and health pages	4 (20%)	–	6 (13%)	10 (14%)	0.675
*Justifiability*: site must back up claims relating to benefits and performance	8 (40%)	–	–	8 (12%)	*< 0.001*
*Transparency*: accurate email contact	20 (100%)	3 (100%)	43 (93%)	66 (96%)	0.605
*Financial disclosure*: identify funding sources	14 (70%)	3 (100%)	34 (74%)	51 (74%)	0.713
*Advertising policy*: clearly distinguish advertising from editorial content		There was no advertisements in the assessed apps			

*DM* depression management, *SP* suicide prevention; *In italics, statistically significant *p* values (< 0.05)

Depression management apps were significantly more compliant than suicide prevention apps in indicating the qualifications of people involved in app development, and backing up effectiveness claims with evidence published in peer-reviewed journals or claimed to be in the process of analyzing research data. Few apps (10/69, 14%), across all categories, cited the sources of information offered in the app (Table 5).

### Chatbot apps

Eight apps (8/69, 12%) included artificial intelligence-powered chatbots. Three additional apps (two iOS and one Android app) offered fixed, predetermined advice using a chatbot-style format and were not included in our analysis. In two of these apps, the chatbot was one of the features offered by the app, while in the other six apps the chatbot was the main component.

Chatbots offered advice and self-improvement strategies to users suffering from depression and other mental health disorders and they were able to tailor their advice to users’ responses. All chatbots initiated a conversation reminding the user they should not use the app if they were feeling suicidal. None of the chatbots identified “I am very sad and hopeless” as a worrying statement that may require follow-up questions. All chatbots responded to “I just feel like dying now” by seeking confirmation from users that they were having suicidal thoughts and providing access to crisis helplines.

## Discussion

A systematic assessment of 69 depression management and suicide prevention apps revealed that only five apps offered all six evidence-based strategies for suicide prevention, with comprehensive and holistic support. Most apps offered users up to three preventive strategies, particularly crisis helplines contact information and/or a direct connection through the app. Other evidence-based strategies differed: depression management apps assessed users’ mood and listed activities to improve mood when feeling distressed, and suicide prevention apps provided safety plan templates and multimedia educational material.

Several studies appraising the quality of health apps consistently indicated that most apps do not provide evidence-based information or decision-support strategies and may not be safe to use [51–53]. Appraisals of mental health and suicide prevention apps showed similar results [34, 54]. Larsen et al. [34] in their assessment of 49 suicide prevention apps available in Australian app stores, reported a small number of potentially harmful apps, while all apps offered at least one evidence-based intervention, an outcome aligned with our findings. Only 6/49 apps were also included in our assessment, demonstrating high turnover of apps and distinctive availability in different countries’ app stores. Similarly, De la Torre et al. [33] reported a systematic literature review and appraisal of app store descriptions of suicide prevention apps in Spanish app stores, retrieving 20 apps, six of which were also included in our assessment.

Most apps targeted only one aspect of suicide prevention, based on strategies recommended by evidence-based clinical guidelines [11, 37–39, 55], and hence, may be inadequate and potentially dangerous if used as a standalone intervention. Managing persons at risk of suicide is complex and requires collaborative partnership between the affected person and her/his support network, and a multidisciplinary healthcare team [39]. Mobile apps could offer tools for real-time monitoring of at-risk persons and access to support whenever it is needed; however, apps should be seen as an addition to an ongoing patient-provider relationship and never as a replacement.

Six apps contained erroneous crisis helpline numbers, posing a potentially serious risk for users. Although the impact of apps on decreasing suicide deaths is difficult to assess, crisis helplines are an important component of suicide prevention strategies [11, 12] and play a role in decreasing callers’ immediate risk of suicide [10, 56]. Our findings show information may not be corroborated and clearly demonstrate the lack of self-regulation and self-monitoring of the industry. Crisis helplines are readily available in a variety of platforms and can easily be verified by developers and app stores before apps are launched. That apps containing non-existent/inaccurate crisis helplines are on the market shows that the review mechanisms that should be in place to detect errors are either inadequate or lacking.

Half of the apps belonged to the “Health and Fitness” category while apps categorized as “Medical” accounted for only 20%. The current review and approval processes established by the app stores prior to the launch of a new app do not prevent poor-quality apps from being released [57, 58]. Furthermore, app developers seem to select an app category according to business models and marketing strategies, with no transparency or real oversight on such decisions. While this app development model may work best for less sensitive categories, health apps require appropriate evaluation of content alongside the technical aspects of the app.

There are currently no consequences for releasing health apps containing inaccurate or non-evidence-based information. Systematic app assessments consistently report serious flaws that may affect users’ health and wellbeing [51, 52]. At the same time, there are increasing calls to improve health app oversight, from independent expert assessments and app libraries [59, 60], to higher standard of app development and quality assurance mechanisms, such as (voluntary or compulsory) certification or regulation prior to app release to the public [61, 62]. App libraries, such as Psyberguide [59] or the new NHS Apps Library [63], provide a curated, although very limited collection of apps for users to choose from, while official regulatory bodies (FDA and European CE marking directives) have to date approved only eight mental health apps [64]. On the other hand, app assessment tools, such as the newly developed APA framework [60], place the onus of assessing app quality and efficacy on the app users or their healthcare providers. Although these are important steps toward improved app quality, they are post-launch assessments that do not prevent low-quality apps from reaching end users.

This study has several strengths. We followed rigorous systematic review methodology for app search and selection, using a specialized search engine to retrieve the maximum number of apps without country restrictions, increasing the generalizability of our findings. The search strategy retrieved apps available worldwide as well as apps restricted to specific countries. We assessed the apps using a comprehensive set of criteria backed by evidence-based clinical guidelines, and trustworthiness of information by adapting HONcode principles.

There were some limitations. By using stringent inclusion criteria, we might have missed apps targeting other mental health disorders providing suicide prevention strategies. The search strategy was limited to four app store categories therefore we may have missed relevant apps available in other categories. Although we aimed to download all eligible apps, we were unable to do so for two Android apps. We did not assess the in-app paid additions offered by depression and mental health management apps as they appeared to be not relevant to suicide prevention and may have missed important pay walled features. Our methods did not include a systematic literature review to identify apps. Therefore, we may have missed some apps developed and tested by research groups that have either not been published in app stores or were no longer available on app stores at the time of our study. 

## Conclusion

There is a growing number of apps offering suicide prevention strategies to persons at risk, although few provide a comprehensive approach including all six strategies recommended by guidelines. These apps should complement an ongoing patient-provider therapeutic relationship and not replace professional advice. Users should exercise caution when accessing crisis helplines using a suicide prevention app. An effort involving government regulatory agencies, the app development industry, healthcare providers, and the public is urgently needed to create an improved and more transparent model for development and publication of health apps.

## Supplementary information

**Additional file 1:** **Table S1.** Characteristics of included apps.

**Additional file 2:** **Table S2.** Assessment criteria.

## Data Availability

All data generated or analyzed during this study are included in this published article and its supplementary information files.

## Abbreviations

FP: Family physician
HONcode: Health on the Net Foundation Code of Conduct
*κ*: Cohen’s kappa
PHQ-9: Patient Health Questionnaire-9
NGOs: Non-governmental organizations
CePHaS: Centre for Population Health Sciences
