# Supplementary material for: Suicide prevention and depression apps’ suicide risk assessment and management: a systematic assessment of adherence to clinical guidelines
Source: BMC Med. 2019 Dec 19;17:231. doi: 10.1186/s12916-019-1461-z (PMC6921471; doi:10.1186/s12916-019-1461-z)
Supplement: Supplementary file 1 — Additional file 1: Table S1. Characteristics of included apps. [file 12916_2019_1461_MOESM1_ESM.docx]

**Supplementary Table 1**: Characteristics of included apps

| **App characteristics** | | | | **App functionalities** | | | | | | | | | | |
| --- | --- | --- | --- | --- | --- | --- | --- | --- | --- | --- | --- | --- | --- | --- |
| **App name** | **Platform** | **App category** | **Developed by** | **Assessment** | | **Safety plan** | | **Activities to deter suicidal thoughts** | **Suicide related information** | **Access to support networks** | **Access to emergency resources** | | | |
|  |  |  |  | **Mood, depression** | **Suicidal thoughts** | **Has safety plan?** | **Shared with support network** |  |  |  | **Emergency contact information** | | **Emergency counselling** | |
|  |  |  |  |  |  |  |  |  |  |  | **Type of information** | **Country specific?** | **In-app counselling** | **Connects to helpline** |
| #LetsTalk  <https://play.google.com/store/apps/details?id=com.app_allianceforyouth.layout&hl=en_SG> | Android | SP | NGO  USA | No | No | No | NA | No | Yes, general information | No | Phone numbers, Text | Yes, USA | No | Yes |
| 2 Calls A Day  (withdrawn) | Android | SP Emergency contact | Healthcare providers USA | No | No | No | NA | No | No | Yes | No | NA | No | No |
| 7 Cups  <https://play.google.com/store/apps/details?id=com.sevencupsoftea.app> | Android | DM  Chatbot | Healthcare providers USA | Yes - PHQ-9 | No | No | NA | No | No | No | Phone numbers | Yes, USA | No | Yes |
| AlachuaTalk  <https://play.google.com/store/apps/details?id=com.t726fbfc8e1b.www> | Android | SP | Gov. Dep. USA | No | No | No | NA | No | Yes, general information | No | Phone numbers | Yes, USA | No | Yes |
| Be Strong  <https://play.google.com/store/apps/details?id=global.bestrong.bestrong&hl=en_SG> | Android | SP  Emergency contact | NGO  USA | No | No | No | NA | No | Yes | Yes, stores family/friends phone numbers | Phone numbers, chats | Yes, USA | No | Yes |
| BeyondNow  <https://play.google.com/store/apps/details?id=au.org.beyondblue.beyondnow&hl=en_SG> | Android | SP  Safety plan | University & NGO Australia | No | No | Yes | Yes | No | Yes, safety plan | Yes, stores numbers in safety plan | Phone numbers | Yes, Australia | No | Yes |
| CBT Mental Health App  <https://play.google.com/store/apps/details?id=mentalhealthapp.mhamzasani.com.mentalhealthapp&hl=en_SG> | Android | DM | Private developers Country unknown | Yes - PHQ-9 | No | No | NA | Yes^a^ | No | No | Phone numbers, Websites | No | No | No |

**Supplementary Table 1**: Characteristics of included apps (continued)

| **App characteristics** | | | | **App functionalities** | | | | | | | | | | |
| --- | --- | --- | --- | --- | --- | --- | --- | --- | --- | --- | --- | --- | --- | --- |
| **App name** | **Platform** | **App category** | **Developed by** | **Assessment** | | **Safety plan** | | **Activities to deter suicidal thoughts** | **Suicide related information** | **Access to support networks** | **Access to emergency resources** | | | |
|  |  |  |  | **Mood, depression** | **Suicidal thoughts** | **Has safety plan?** | **Shared with support network** |  |  |  | **Emergency contact information** | | **Emergency counselling** | |
|  |  |  |  |  |  |  |  |  |  |  | **Type of information** | **Country specific?** | **In-app counselling** | **Connects to helpline** |
| Change Scope  <https://play.google.com/store/apps/details?id=com.obhoy.changescope&hl=en_SG> | Android | SP | Private developer Bangladesh | Yes | Yes | No | NA | Yes | Yes, general information | No | Phone numbers, addresses of drug rehab centers | Yes, Bangladesh | No | No |
| DBT Distress Tolerance Tools  <https://play.google.com/store/apps/details?id=com.poppop.DBTDistressTolerance&hl=en_SG> | Android | DM | Private developer USA | No | No | No | NA | No | No | No | Phone numbers, Websites | Yes, USA | No | Yes |
| iWorth  <https://play.google.com/store/apps/details?id=com.app.iworth&hl=en_SG> | Android | DM | Private developers Country unknown | No | No | No | NA | Yes^a^ | No | No | Phone numbers, Websites | No | No | No |
| Just In Case for Colleges  <https://play.google.com/store/apps/details?id=com.ereadia.justincase2&pcampaignid> | Android | SP | Gov. Dep. USA | No | No | No | NA | No | Yes | No | Phone numbers | Yes, USA | No | Yes |
| Kokua Life  (withdrawn) | Android | SP | NGO  USA | Yes - PHQ-9 | Yes | Yes | No | Yes | Yes, general information & safety plan | No | Phone numbers, text, websites | Yes, USA | No | Yes |
| Moodpath  <https://play.google.com/store/apps/details?id=de.moodpath.android> | Android | DM | Healthcare providers Germany | Yes | Yes | No | NA | Yes^a^ | No | No | Helpline (country specific) | No | No | Yes |
| MoodTools  <https://play.google.com/store/apps/details?id=com.moodtools.moodtools> | Android | DM | Healthcare providers USA | Yes - PHQ-9 | No | Yes | No | No | Yes, safety plan | Yes, stores family/friends phone number in safety plan | Web, Google map locations | No - but Google map gives area-tailored info | No | No |

**Supplementary Table 1**: Characteristics of included apps (continued)

| **App characteristics** | | | | **App functionalities** | | | | | | | | | | |
| --- | --- | --- | --- | --- | --- | --- | --- | --- | --- | --- | --- | --- | --- | --- |
| **App name** | **Platform** | **App category** | **Developed by** | **Assessment** | | **Safety plan** | | **Activities to deter suicidal thoughts** | **Suicide related information** | **Access to support networks** | **Access to emergency resources** | | | |
|  |  |  |  | **Mood, depression** | **Suicidal thoughts** | **Has safety plan?** | **Shared with support network** |  |  |  | **Emergency contact information** | | **Emergency counselling** | |
|  |  |  |  |  |  |  |  |  |  |  | **Type of information** | **Country specific?** | **In-app counselling** | **Connects to helpline** |
| MYPLAN  <https://play.google.com/store/apps/details?id=english.minplan.controllers> | Android | SP  Safety plan | Private developer Norway | No | No | Yes | No | Yes | Yes, general information | Yes, stores numbers in safety plan | Phone numbers, addresses (user needs to input the information) | No | No | Yes |
| notOK  <https://play.google.com/store/apps/details?id=com.robinlucas.notok&hl=en_SG> | Android | SP  Emergency contact | Private developer USA | No | No | No | NA | No | No | Yes | No | NA | No | No |
| Operation Life  <https://play.google.com/store/apps/details?id=operationlife.dva.com.operationlife&hl=en> | Android | SP | Gov. Dep. Australia | No | No | No | NA | Yes | Yes, general information | Yes | Phone numbers, websites | Yes, Australia | Yes | No |
| Pacifica  (now Sanvello)  <https://play.google.com/store/apps/details?id=com.pacificalabs.pacifica&hl=en> | Android | DM | Private developer USA | Yes | No | No | NA | Yes^a^ | No | Yes, stores provider phone number | Phone numbers, Websites | No | No | Yes |
| Prevent Suicide – Highland  <https://play.google.com/store/apps/details?id=com.faffdigital.PSHighland&hl=en> | Android | SP  Safety plan | Gov. Dep. Scotland | No | No | Yes | No | No | Yes, general information & safety plan | No | Phone numbers, websites | Yes, Scotland & UK | No | Yes |
| Prevent Suicide - NE Scotland  <https://play.google.com/store/apps/details?id=com.faffdigital.PreventSuicide&hl=en> | Android | SP  Safety plan | Gov. Dep. Scotland | No | No | Yes | No | No | Yes, general information & safety plan | No | Phone numbers, websites | Yes - Scotland & UK | No | Yes |

**Supplementary Table 1**: Characteristics of included apps (continued)

| **App characteristics** | | | | **App functionalities** | | | | | | | | | | |
| --- | --- | --- | --- | --- | --- | --- | --- | --- | --- | --- | --- | --- | --- | --- |
| **App name** | **Platform** | **App category** | **Developed by** | **Assessment** | | **Safety plan** | | **Activities to deter suicidal thoughts** | **Suicide related information** | **Access to support networks** | **Access to emergency resources** | | | |
|  |  |  |  | **Mood, depression** | **Suicidal thoughts** | **Has safety plan?** | **Shared with support network** |  |  |  | **Emergency contact information** | | **Emergency counselling** | |
|  |  |  |  |  |  |  |  |  |  |  | **Type of information** | **Country specific?** | **In-app counselling** | **Connects to helpline** |
| Safety Plan  <https://play.google.com/store/apps/details?id=com.blue_bird_tech.safetyplan.safetyplan&hl=en> | Android | SP  Safety plan | Private developers Country unknown | No | No | Yes | No | Yes | No | Yes, stores numbers in safety plan | Phone numbers | Yes, USA (user can add own) | No (user can contact provider from safety plan) | Yes (user can edit it) |
| SafetyNet  <https://play.google.com/store/apps/details?id=com.application.safetynet&hl=en> | Android | SP  Safety plan | Private developers Canada | No | No | Yes | No | Yes | No | Yes, stores numbers in safety plan | Phone numbers, websites | Yes, North America, UK & Australia | No (user can contact provider from safety plan) | Yes |
| SafeUT  <https://play.google.com/store/apps/details?id=com.p3tips.safeut&hl=en_SG> | Android | SP  Emergency contact | University USA | No | No | No | NA | No | No | No | Phone and chat numbers | Yes, USA | Yes | Yes |
| SOS – SendOutSupport  (withdrawn) | Android | SP  Emergency contact | Private developer Canada | No | No | No | NA | Yes | Yes, general information | Yes | Phone numbers, websites | No | No | Yes (user needs to edit the number) |
| Stay Alive  <https://play.google.com/store/apps/details?id=uk.org.suicideprevention.stayalive&hl=en_SG> | Android | SP  Safety plan | NGO  UK | No | No | Yes | No | Yes | Yes, general information | Yes | Phone numbers, websites | Yes, UK | No | Yes |
| Suicide Safety Plan  <https://play.google.com/store/apps/details?id=com.moodtools.crisis.app&hl=en_SG> | Android | SP  Safety plan | Healthcare providers USA | No | No | Yes | No | Yes | Yes, general information & safety plan | Yes, stores numbers in safety plan | Phone numbers, websites, guides | No | No (user can contact provider from safety plan) | No |
| Suicide? Help!  <https://play.google.com/store/apps/details?id=com.faffdigital.ChooseLife&hl=en_SG> | Android | SP  Safety plan | Gov. Dep. Scotland | No | No | Yes | No | No | Yes, general information & safety plan | No | Phone numbers, websites | Yes, Scotland & UK | No | Yes |

**Supplementary Table 1**: Characteristics of included apps (continued)

| **App characteristics** | | | | **App functionalities** | | | | | | | | | | |
| --- | --- | --- | --- | --- | --- | --- | --- | --- | --- | --- | --- | --- | --- | --- |
| **App name** | **Platform** | **App category** | **Developed by** | **Assessment** | | **Safety plan** | | **Activities to deter suicidal thoughts** | **Suicide related information** | **Access to support networks** | **Access to emergency resources** | | | |
|  |  |  |  | **Mood, depression** | **Suicidal thoughts** | **Has safety plan?** | **Shared with support network** |  |  |  | **Emergency contact information** | | **Emergency counselling** | |
|  |  |  |  |  |  |  |  |  |  |  | **Type of information** | **Country specific?** | **In-app counselling** | **Connects to helpline** |
| Tennessee Suicide Prevention Network  <https://play.google.com/store/apps/details?id=com.tspn&hl=en_SG> | Android | SP | NGO  USA | No | No | No | NA | No | Yes, general information | No | Phone and chat numbers | Yes, USA | No | Yes |
| There Is Hope 2  <https://play.google.com/store/apps/details?id=com.nextlogik.grassroots&hl=en> | Android | SP  Safety plan | NGO  USA | No | Yes | Yes | No | No | Yes, general information & safety plan | No | Phone numbers | Yes, USA | No | Yes |
| TUFMinds  <https://play.google.com/store/apps/details?id=com.app_tufminds.layout&hl=en> | Android | DM & SP | Healthcare provider Australia | No | No | No | No | No | Yes, general information | No | Phone numbers | No | No | No |
| WellMind  <https://play.google.com/store/apps/details?id=com.bluestepsolutions.wellmind&hl=en> | Android | DM | Gov. Dep. UK | Yes | No | No | NA | Yes^a^ | No | No | Phone numbers | Yes, UK & Ireland | No | No |
| Woebot  <https://play.google.com/store/apps/details?id=com.woebot&hl=en> | Android | DM  Chatbot | Healthcare providers USA | Yes | Yes | No | NA | Yes^a^ | No | No | Phone numbers | Yes, USA | No | No |
| Wysa  <https://play.google.com/store/apps/details?id=bot.touchkin&hl=en> | Android | DM  Chatbot | Private developer UK/ India | Yes | Yes | No | NA | Yes^a^ | No | No | Phone numbers, chat lines | No | No | Yes |
| Youper  <https://play.google.com/store/apps/details?id=br.com.youper&hl=en> | Android | DM  Chatbot | Healthcare providers USA | Yes | Yes | No | NA | Yes^a^ | No | No | Phone numbers, websites | No | No | No |

**Supplementary Table 1**: Characteristics of included apps (continued)

| **App characteristics** | | | | **App functionalities** | | | | | | | | | | |
| --- | --- | --- | --- | --- | --- | --- | --- | --- | --- | --- | --- | --- | --- | --- |
| **App name** | **Platform** | **App category** | **Developed by** | **Assessment** | | **Safety plan** | | **Activities to deter suicidal thoughts** | **Suicide related information** | **Access to support networks** | **Access to emergency resources** | | | |
|  |  |  |  | **Mood, depression** | **Suicidal thoughts** | **Has safety plan?** | **Shared with support network** |  |  |  | **Emergency contact information** | | **Emergency counselling** | |
|  |  |  |  |  |  |  |  |  |  |  | **Type of information** | **Country specific?** | **In-app counselling** | **Connects to helpline** |
| #LetsTalk  <https://apps.apple.com/app/letstalk/id1300758165> | iOS | SP | NGO  USA | No | No | No | NA | No | Yes, general information | No | Phone numbers, Text, "safe places" addresses | Yes, USA | No | Yes |
| 2 Calls A Day  <https://apps.apple.com/app/2callsaday/id1244118224> | iOS | SP  Emergency contact | Healthcare providers USA | No | No | No | NA | No | No | Yes | No | No | No | No |
| 7 Cups  <https://apps.apple.com/us/app/7-cups-anxiety-stress-chat/id921814681> | iOS | DM  Chatbot | Healthcare providers USA | Yes - PHQ-9 | No | No | NA | No | No | No | Phone numbers | Yes, USA | No | Yes |
| A.L.E.R.T.  <https://apps.apple.com/app/a-l-e-r-t/id674020842> | iOS | DM & SP | NGOs Australia | Yes - PHQ-2 | No | No | NA | Yes | Yes, general information | Yes | Phone numbers, websites | Yes, Australia | No | Yes |
| Be Safe  <https://apps.apple.com/nz/app/be-safe/id1372439182> | iOS | SP  Safety plan | Private developer Australia | No | No | Yes | No | No | No | No | Phone numbers | Yes, Australia | No | Yes |
| Be Strong  <https://apps.apple.com/us/app/be-strong/id1148602800> | iOS | SP  Emergency contact | NGO  USA | No | No | No | NA | No | Yes | Yes - Family/ friends phone numbers | Phone numbers, chats | Yes, USA | No | Yes |
| BeyondNow  <https://apps.apple.com/au/app/beyondnow-suicide-safety-plan/id1059270058> | iOS | SP  Safety plan | University & NGO Australia | No | No | Yes | Yes | No | Yes, safety plan | Yes, stores numbers in safety plan | Phone numbers | Yes, Australia | No | Yes |

**Supplementary Table 1**: Characteristics of included apps (continued)

| **App characteristics** | | | | **App functionalities** | | | | | | | | | | |
| --- | --- | --- | --- | --- | --- | --- | --- | --- | --- | --- | --- | --- | --- | --- |
| **App name** | **Platform** | **App category** | **Developed by** | **Assessment** | | **Safety plan** | | **Activities to deter suicidal thoughts** | **Suicide related information** | **Access to support networks** |  | | | |
|  |  |  |  | **Mood, depression** | **Suicidal thoughts** | **Has safety plan?** | **Shared with support network** |  |  |  | **Emergency contact information** | | **Emergency counselling** | |
|  |  |  |  |  |  |  |  |  |  |  | **Type of information** | **Country specific?** | **In-app counselling** | **Connects to helpline** |
| DBT Distress Tolerance Tools  <https://apps.apple.com/us/app/dbt-distress-tolerance-tools/id955926299> | iOS | DM | Private developer (USA) | No | No | No | NA | No | No | No | Phone numbers, Websites | Yes, USA | No | Yes |
| DMHS  <https://apps.apple.com/app/dmhs-interactive-suicide-prevention/id1136542675> | iOS | SP  Safety plan | Healthcare providers  Canada | Yes | Yes | Yes | Yes | Yes | Yes | Yes | Phone number | Yes, Canada | Yes | No |
| First Step OR.  <https://apps.apple.com/app/first-step-or/id1332019378> | iOS | SP | Gov. Dep. USA | No | No | No | NA | Yes | No | No | Phone numbers, websites | Yes, USA | No | Yes |
| Guard Your Buddy – Tennessee  <https://apps.apple.com/app/guard-your-buddy-tennessee/id484289738> | iOS | SP | NGO  USA | No | No | No | NA | No | Yes, general information | No | Phone number, email address | Yes, USA | Yes | No |
| Help Is Here  <https://apps.apple.com/app/help-is-here/id1387303972> | iOS | SP | University ? NGO  USA | No | No | No | NA | No | Yes, general information | Yes | Phone and chat numbers | Yes, USA | Yes | Yes |
| Just in Case  <https://apps.apple.com/app/just-in-case/id1291684209> | iOS | SP | Gov. Dep. USA | No | No | No | NA | No | Yes, general information | No | Phone numbers | Yes, USA | No | Yes |
| Kokua Life  <https://apps.apple.com/app/kokua-life/id1237803419> | iOS | SP | NGO  USA | Yes - PHQ-9 | Yes | No | NA | Yes | Yes, general information & safety plan | No | Phone numbers, text, websites | Yes, USA | No | Yes |

**Supplementary Table 1**: Characteristics of included apps (continued)

| **App characteristics** | | | | **App functionalities** | | | | | | | | | | |
| --- | --- | --- | --- | --- | --- | --- | --- | --- | --- | --- | --- | --- | --- | --- |
| **App name** | **Platform** | **App category** | **Developed by** | **Assessment** | | **Safety plan** | | **Activities to deter suicidal thoughts** | **Suicide related information** | **Access to support networks** | **Access to emergency resources** | | | |
|  |  |  |  | **Mood, depression** | **Suicidal thoughts** | **Has safety plan?** | **Shared with support network** |  |  |  | **Emergency contact information** | | **Emergency counselling** | |
|  |  |  |  |  |  |  |  |  |  |  | **Type of information** | **Country specific?** | **In-app counselling** | **Connects to helpline** |
| Moodpath  <https://apps.apple.com/app/moodpath-depression-anxiety/id1052216403> | iOS | DM | Healthcare providers Germany | Yes | Yes | No | NA | Yes^a^ | No | No | Helpline (country specific) | Yes | No | Yes |
| MoodTools  <https://apps.apple.com/app/moodtools-depression-aid/id1012822112> | iOS | DM | Healthcare providers USA | Yes - PHQ-9 | No | Yes | No | Yes^a^ | Yes, safety plan | Yes, stores family/friends phone number in safety plan | Phone numbers, Websites | Yes, USA | No | Yes |
| notOK  <https://apps.apple.com/app/notok/id1322629109> | iOS | SP  Emergency contact | Private developer USA | No | No | No | NA | No | No | Yes | No | NA | No | No |
| Operation Life  <https://apps.apple.com/app/operation-life/id1030670665> | iOS | SP | Gov. Dep. Australia | No | No | No | NA | Yes | Yes, general information | Yes | Phone numbers | Yes, Australia | Yes | Yes |
| Pacifica  (now Sanvello)  <https://apps.apple.com/app/pacifica-for-stress-anxiety/id922968861> | iOS | DM | Private developer USA | Yes | No | No | NA | Yes^a^ | No | No, can only email report to user's network | Phone numbers, Websites | No | No | Yes |
| Prevent Suicide – Highland  <https://apps.apple.com/app/prevent-suicide-highland/id1436026798> | iOS | SP  Safety plan | Gov. Dep. Scotland | No | No | Yes | No | No | Yes, general information & safety plan | No | Phone numbers, websites | Yes, Scotland & UK | No | Yes |
| Prevent Suicide  <https://apps.apple.com/app/prevent-suicide/id1071088093> | iOS | SP  Safety plan | Gov. Dep. Scotland | No | No | Yes | No | No | Yes, general information & safety plan | No | Phone numbers, websites | Yes, Scotland & UK | No | Yes |

**Supplementary Table 1**: Characteristics of included apps (continued)

| **App characteristics** | | | | **App functionalities** | | | | | | | | | | |
| --- | --- | --- | --- | --- | --- | --- | --- | --- | --- | --- | --- | --- | --- | --- |
| **App name** | **Platform** | **App category** | **Developed by** | **Assessment** | | **Safety plan** | | **Activities to deter suicidal thoughts** | **Suicide related information** | **Access to support networks** | **Access to emergency resources** | | | |
|  |  |  |  | **Mood, depression** | **Suicidal thoughts** | **Has safety plan?** | **Shared with support network** |  |  |  | **Emergency contact information** | | **Emergency counselling** | |
|  |  |  |  |  |  |  |  |  |  |  | **Type of information** | **Country specific?** | **In-app counselling** | **Connects to helpline** |
| ReliefLink  <https://apps.apple.com/app/relieflink/id721474553> | iOS | SPSafety plan | University USA | Yes | Yes | Yes | No | Yes | No | Yes | Phone numbers, websites | No (user can add own) | No | Yes |
| ReMinder App  <https://apps.apple.com/au/app/reminder-app/id1407116715> | iOS | SP  Safety plan | NGO Australia | Yes - K-10 | No | Yes | No | Yes | No | Yes | Phone numbers | Yes, Australia | Yes | Yes |
| SafeUT  <https://apps.apple.com/app/safeut/id1052510262> | iOS | SP  Emergency contact | University USA | No | No | No | NA | No | No | No | No | NA | Yes | Yes |
| Stanley-Brown Safety Plan  <https://apps.apple.com/app/stanley-brown-safety-plan/id695122998> | iOS | SP  Safety plan | Gov.Dep. USA | No | No | Yes | Yes | Yes | No | Yes | Phone numbers, websites | Yes, USA | No | Yes |
| Stay Alive  <https://apps.apple.com/app/stay-alive/id915458967> | iOS | SP  Safety plan | NGO  UK | Yes | No | Yes | No | Yes | Yes, general information | No, just saves names | Phone numbers, websites | Yes, UK | No | Yes |
| StayAfloat  <https://apps.apple.com/app/stayafloat/id1255192613> | iOS | SP  Emergency contact | Private developer USA | No | No | No | NA | No | No | Yes | Phone number | No (user can add own) | No | Yes |
| Suicide Safety Plan  <https://apps.apple.com/app/suicide-safety-plan/id1003891579> | iOS | SP  Safety plan | Healthcare providers USA | No | No | Yes | No | Yes | Yes, general information & safety plan | Yes, stores numbers in safety plan | No | No | No | No |
| Suicide? Help!  <https://apps.apple.com/app/suicide-help/id575896482> | iOS | SP  Safety plan | Gov. Dep. Scotland | No | No | Yes | No | No | Yes, general information & safety plan | No | Phone numbers, websites | Yes, Scotland & UK | No | Yes |

**Supplementary Table 1**: Characteristics of included apps (continued)

| **App characteristics** | | | | **App functionalities** | | | | | | | | | | |
| --- | --- | --- | --- | --- | --- | --- | --- | --- | --- | --- | --- | --- | --- | --- |
| **App name** | **Platform** | **App category** | **Developed by** | **Assessment** | | **Safety plan** | | **Activities to deter suicidal thoughts** | **Suicide related information** | **Access to support networks** | **Access to emergency resources** | | | |
|  |  |  |  | **Mood, depression** | **Suicidal thoughts** | **Has safety plan?** | **Shared with support network** |  |  |  | **Emergency contact information** | | **Emergency counselling** | |
|  |  |  |  |  |  |  |  |  |  |  | **Type of information** | **Country specific?** | **In-app counselling** | **Connects to helpline** |
| There Is Hope  <https://apps.apple.com/app/there-is-hope/id1122136102> | iOS | SP  Safety plan | NGO  USA | Yes | Yes | Yes | No | No | Yes, general information & safety plan | No | Phone numbers | Yes, USA | No | Yes |
| TUFMinds  <https://apps.apple.com/app/tufminds/id1439734182> | iOS | DM & SP | Healthcare provider Australia | No | No | No | No | No | Yes, general information | No | Phone numbers, websites | Yes, Australia, NZ, UK, USA | No | Yes |
| WellMind  <https://apps.apple.com/app/wellmind/id918138339> | iOS | DM | Gov. Dep. UK | Yes | No | No | NA | Yes^a^ | No | No | Phone numbers | Yes, UK & Ireland | No | No |
| Woebot  <https://apps.apple.com/app/woebot-depression-anxiety/id1305375832> | iOS | DM  Chatbot | Healthcare providers USA | Yes | Yes | No | NA | Yes | No | No | Phone numbers | Yes, USA | No | No |
| Wysa  <https://apps.apple.com/app/wysa-anxiety-depression-bot/id1166585565> | iOS | DM  Chatbot | Private developer UK/ India | Yes | Yes | No | NA | Yes | No | No | Phone numbers, chat lines | No | No | Yes |
| Youper - Ai Mindfulness  <https://apps.apple.com/app/youper-ai-mindful-therapy/id1060691513> | iOS | DM  Chatbot | Healthcare providers USA | Yes | Yes | No | NA | Yes | No | No | Phone numbers, websites | Yes | No | No |

DM: Depression Management; SP: Suicide Prevention; NGO: Non-governmental organization; Gov. Dep: Government Department

^a^Provides relaxing activities, not exclusively as suicide-deterrent
