# Supplementary material for: Suicide prevention and depression apps’ suicide risk assessment and management: a systematic assessment of adherence to clinical guidelines
Source: BMC Med. 2019 Dec 19;17:231. doi: 10.1186/s12916-019-1461-z (PMC6921471; doi:10.1186/s12916-019-1461-z)
Supplement: Supplementary file 2 — Additional file 2: Table S2. Assessment criteria. [file 12916_2019_1461_MOESM2_ESM.docx]

**Supplementary Table 2**: Assessment criteria

| Field | Ref | Description | Options | Justification/Evidence |
| --- | --- | --- | --- | --- |
| 1. App Description (from App Store) |  |  |  |  |
| App ID | 1-1 | A unique ID number generated for each app for easy identification and merging of data. | Alpha-numeric |  |
| App Name | 1-2 | The given name/title of the app. | Free text |  |
| Developer name | 1-3 | The company of the app developer. If there is no name, note the developer’s name. | Free text |  |
| Platform | 1-4 | Is this app tested on Android or iOS? | Android/iOS |  |
| App version number | 1-5 | List the latest version of the app during assessment | Alpha-numeric |  |
| User rating | 1-6 | State user ratings if applicable on a scale of 5. | Numeric |  |
| Number of people who rated | 1-7 | State the number of people who provided ratings for the app at the point of assessment. | Numeric |  |
| Cost (App or in-app purchases) | 1-8 | Cost to download the app or extra features. | Numeric |  |
| Language | 1-9-1 | What is the primary language of the app? | English/Chinese/Spanish/Others |  |
|  | 1-9-2 | What language is the app being assessed? | English/Chinese/Spanish/Others |  |
| Accessible region | 1-10 | Is this app limited to certain locations/ countries/ regions? | Free text |  |
| Target user | 1-11 | Who is the target user of the app? | Adult Patient/ All |  |
| Scope of the app | 1-12 | What kind of app is it? | Mental Health/Depression/ Suicide/Depression with Suicide advice/Chatbot/All/Other |  |
| Tracking of condition status | 1-13 | Does the app have any function related to tracking of mood (e.g. hopelessness, desire to die, others), triggers, protective factors, safety plans, others? | Yes/No |  |
| Safety plan | 1-14 | Does the app allows user to develop/ store a safety plan? | Yes/No |  |
| Self-harm deterring activities | 1-15 | Does the app provide activities to the user to deter the urge of self-harm? | Yes/No |  |
| Information & education | 1-16 | Does the app provide information on suicide risk factors (psychological or environmental), safety plan, emergency management? | Yes/No |  |

**Supplementary Table 2**: Assessment criteria (continued)

| Field | Ref | Description | Options | Justification/Evidence |
| --- | --- | --- | --- | --- |
| Access to support networks | 1-17 | Does the app allow the user to share information or contact people in her/his support network? (e.g. family members, friends, healthcare providers, others) | Yes/No |  |
| Emergency counselling | 1-18-1 | Does the app allow contact with professional help? | Yes/No |  |
|  | 1-18-2 | Does the app allow contact with non-professional, non-familiar support network? (forums, 1-on-1 chats, buddies, etc…) | Yes/No |  |
| 2. Tracking of condition status |  |  |  |  |
| 2-1. Tracking of mood status | 2-1-1 | Does the app enquire about feelings of hopelessness, impulsiveness, anhedonia, panic attacks, or anxiety? | Yes/No/NA | Any patient with the following conditions should be assessed and managed using this guideline: a. Person ... (with) mental disorders (Depression, bipolar, schizophrenia, PTSD), or medical condition (TBI, pain, sleep disturbance) ... c. Person scores very high on depression screening tool ... d. Person reports suicidal thoughts on depression screening tool e. Woman reports suicidal thoughts on depression screening tool during pregnancy or postpartum visits.... (VA guideline 2013) |
|  | 2-1-2 | Does the app administer any questionnaire to assess risk of depression, anxiety or other mental disorder? | Yes/No/NA |  |
|  | 2-1-3 | Does the app enquire about alcohol or other substance use associated with the current presentation? | Yes/No/NA |  |
|  | 2-1-4 | Does the app enquire about user's ability to tolerate psychological pain? | Yes/No/NA |  |
| 2-2. Tracking of suicidal behavior | 2-2-1 | Does the app assess for suicidal or self-harming thoughts, plans, behaviors, and intent? | Yes/No/NA | 1.1.4.6 Always ask people with depression directly about suicidal ideation and intent. (NICE 2009) 1.13 Health care providers should .... be specifically asked about suicide and, if it is a possibility, health care providers must limit the amount of medicines prescribed ... (WHO 2009) Assess the patient’s thoughts of suicide, the intention to act on those thoughts, and behaviors that demonstrate warning signs. (VA guideline 2013) |
|  | 2-2-2 | Does the app enquire about specific methods considered for suicide? | Yes/No/NA |  |
|  | 2-2-3 | Does the app enquire about previous suicide attempt episodes? | Yes/No/NA |  |

**Supplementary Table 2**: Assessment criteria (continued)

| Field | Ref | Description | Options | Justification/Evidence |
| --- | --- | --- | --- | --- |
| 2-3. Tracking of triggers | 2-3-1 | Does the app enquire about acute psychosocial crises and chronic psychosocial stressors? (e.g. actual or perceived interpersonal losses, family discord, domestic violence, past or current sexual or physical abuse or neglect) | Yes/No/NA | All people ... should be offered an assessment of ... the social, psychological and motivational factors specific to the act of self-harm, current suicidal intent and hopelessness, as well as a full mental health and social needs assessment. All people ... should be assessed for risk: ... should include identification of the main clinical and demographic features known to be associated with risk of further self-harm and/or suicide, and identification of the key psychological characteristics associated with risk, in particular depression, hopelessness and continuing suicidal intent. (NICE 2004) |
|  | 2-3-2 | Does the app enquire about employment status, financial difficulties or changes in socioeconomic status? | Yes/No/NA |  |
|  | 2-3-3 | Does the app enquire about quality of family relationships? | Yes/No/NA |  |
|  | 2-3-4 | Does the app enquire about previous or current medical diagnoses and treatments, including surgeries, hospitalizations, or chronic pain? | Yes/No/NA |  |
| 2-4. Tracking of protective factors | 2-4-1 | Does the app enquire about reasons for living and plans for the future? (e.g. current pregnancy, children, positive family relationships, others) | Yes/No/NA | The suicide risk assessment should then include consideration of risk and protective factors that may increase or decrease the patient’s risk of suicide. (VA guideline 2013) |
|  | 2-4-2 | Does the app enquire about the user's coping or problem-solving skills? | Yes/No/NA |  |
| 3. Safety plan |  |  |  |  |
| 3-1. Safety plan development | 3-1-1 | Does the app let the user develop a safety plan? | Yes/No/NA | Establish an individualized Safety Plan for all persons who are at high acute risk for suicide as part of discharge planning, regardless of inpatient or outpatient status. The Safety Plan is designed to empower the patient, manage the suicidal crisis, and engage other resources. Discuss safety with patients at intermediate and low risk and consider offering education about safety, and a copy of a Safety Plan handout. (VA guideline 2013) |
|  | 3-1-2 | If yes, does the app provides guidance? | Yes/ No/NA |  |
|  | 3-1-3 | If yes, how does the app do so? (questionnaires/ write-up template/ others) | Free text |  |
| 3-2. Access to previously developed safety plan | 3-2-1 | Does the app allow user to upload a previously developed safety plan? | Yes/ No/NA |  |

**Supplementary Table 2**: Assessment criteria (continued)

| Field | Ref | Description | Options | Justification/Evidence |
| --- | --- | --- | --- | --- |
| 3-3. Sharing safety plan | 3-3-1 | Does the app allow the user to share the safety plan with primary physician/ family member/ friends/others? | Yes/ No/NA |  |
|  | 3-3-2 | If yes, how does the app accomplish this? (e.g. invitation via email/ shared login to app/ others) | Free text |  |
|  | 3-3-3 | Who does the app share the safety plan with? | Free text |  |
| 4. Self-Harm deterring activities |  |  |  |  |
| 4-1. List activities | 4-1-1 | Does the app provide the user with activities to help deter the urge of self-harm? | Yes/No/NA |  |
|  | 4-1-2 | If yes, what kind of activities does the app suggest? | Mindfulness exercises/ Engage in a hobby/ Contact friend/family/ Others (specify) |  |
| 4-2. Follow-up on outcomes | 4-2-1 | Does the app check if person feels better after trying the activity? | Yes/No/NA |  |
|  | 4-2-2 | If no, does the app suggest a new activity? | Yes/No/NA |  |
|  | 4-2-3 | If no, does the app suggest contacting a helpline or person in the user's support group? | Yes/No/NA |  |
| 5. Information & education |  |  |  |  |
| 5-1. Information on signs of suicidality | 5-1-1 | Does the app provides information on how to detect a person is having suicidal thoughts? | Yes/No/NA |  |
| 5-2. Information on suicide risk factors/ triggers | 5-2-1 | Does the app provide information on risk factors for suicidal thoughts? | Yes/No/NA | Health care professionals should provide adults and their families/caregivers/command, if appropriate, with education regarding suicide, stigma, treatment options, and management strategies. (VA guideline 2013) ...psychiatric management may include encouraging treatment adherence and providing education to the patient and, when indicated, family members and significant others. (APA guideline 2010) |
|  | 5-2-2 | Does the app provide information on what triggers suicidal thoughts | Yes/No/NA |  |
| 5-3. Information on safety plan | 5-3-1 | Does the app provide information on what it is a safety plan? | Yes/No/NA |  |
|  | 5-3-2 | Does the app provide information on how to use the safety plan? | Yes/No/NA |  |

**Supplementary Table 2**: Assessment criteria (continued)

| Field | Ref | Description | Options | Justification/Evidence |
| --- | --- | --- | --- | --- |
| 5-4. Emergency contact information | 5-4-1 | Does the app provide emergency contact information? | Yes/No/NA |  |
|  | 5-4-2 | What kind of information is available? (e.g. phone numbers/ websites/ support groups information/ general information on contacting relevant people in case of need) | Phone numbers/ websites/ support groups information/ general information on contacting relevant people in case of need/ others (please specify) |  |
|  | 5-4-3 | Is the information country/ area specific? | Yes/No/NA |  |
| 6. Access to support networks |  |  |  |  |
| 6-1. Access to user's support network | 6-1-1 | Does the app allow user to store support network people (friends/ family/ others) phone number for immediate call if needed? | Yes/No/NA | 1.1.4.6 ... If there is a risk of self-harm or suicide: • assess whether the person has adequate social support and is aware of sources of help (NICE 2009) 1.13 Health care providers ..... should also construct a regimen in which there is frequent clinical monitoring and also monitoring by family members and friends. (WHO 2009) The presence of a social support system is another factor that may reduce suicide risk (399, 400). Consequently, communicating with members of the patient’s support network may be important in assessing and helping to strengthen social supports (APA guideline 2010) |
|  | 6-1-2 | Does the app allow user to store physician/ therapist phone number for immediate call if needed? | Yes/No/NA |  |
| 6-2. Sharing information | 6-2-1 | Does the app allow the user to share information with family/ friends? | Yes/No/NA |  |
|  | 6-2-2 | Does the app allow the user to share information with primary physician? | Yes/No/NA |  |
|  | 6-2-3 | Does the app provide access to support groups? | Yes/No/NA |  |
|  | 6-2-3-1 | Do peers/buddies/support group respond on message request? | Yes/No/NA |  |
|  | 6-2-3-2 | If yes, how long does it take them to respond? | time in mins |  |
| 7. Emergency counselling |  |  |  |  |
| 7-1. Emergency counselling | 7-1-1 | Does the app provide access to professional advice if user is suicidal? | Yes/No/NA | 1.3.2.1 If a person with depression presents considerable immediate risk to themselves or others, refer them urgently to specialist mental health services. (NICE 2009) When patients are identified in primary care with intermediate or high acute risk for suicide they should be evaluated by behavioral health providers (VA guideline 2013) |

**Supplementary Table 2**: Assessment criteria (continued)

| Field | Ref | Description | Options | Justification/Evidence |
| --- | --- | --- | --- | --- |
|  | 7-1-1-1 | Is the counselling provided by the app or through a link to a third party provider? | Provided by app/ Third party/ NA |  |
|  | 7-1-1-2 | Are the professional credentials of the providers offering advice available to users? | Yes/No/NA |  |
|  | 7-1-1-3 | If yes, does the user need to make an in-app purchase in order to access it? | Yes/No/NA |  |
|  | 7-1-1-4 | If yes, How much is the cost? (include currency as stated in the app) | Alpha-numeric |  |
|  | 7-1-2 | Does the app provide chatbot-based counselling if user is suicidal? | Yes/No/NA |  |
|  | 7-1-2-1 | Does the chatbot understand the opening phrase "I am very sad" as worrisome? | Yes/No/NA |  |
|  | 7-1-2-2 | What does the chatbot replies to the phrase "I just feel like dying now"? | Free text - Please save screenshots of the conversation with the chatbot |  |
|  | 7-1-2-3 | What is the action taken by the chatbot after assessing suicidal risk? | Provides counselling/ Provides suicide hotline phone number/ Provides link to a mental health provider/ Automatically message user's contacts (if uploaded by user previously)/ Others (free text) |  |
|  | 7-1-3 | Does the app directly connects the user with emergency hotlines? | Yes/No |  |
| 8. Other attributes |  |  |  |  |
|  | 8-1 | Can the app create user profiles? | Yes/No |  |
|  | 8-2 | Does the app allow for user to remain anonymous while using it? | Yes/No | Desirable feature given the high degree of stigma associated with mental disease and suicide |
|  | 8-3 | Is the account password protected? | Yes/No | Desirable functionality for privacy to personal health information. |
|  | 8-4 | Does the app provide user with rewards for completing tasks/ education/ condition monitoring? | Yes/No | Desirable functionality to encourage patients to maintain adherence to treatment regimen. |
|  | 8-4-1 | If yes, elaborate on what is being rewarded | Free text |  |
|  | 8-4-2 | If yes, elaborate on the type of reward (e.g. Stars, motivational messages, etc.) | Free text |  |

**Supplementary Table 2**: Assessment criteria (continued)

| Field | Ref | Description | Options | Justification/Evidence |
| --- | --- | --- | --- | --- |
|  | 8-5 | Does the app contain any games? | Yes/No |  |
|  | 8-5-1 | If yes, what is/are the purpose of the game(s)? | Free text |  |
|  | 8-6 | Does the app gets stuck, crash or frozen more than once when using? (Answer "Yes" if app hangs for more than 3s in two occasions) | Yes/No | App will frustrate user if it keeps hanging. |
|  | 8-7 | Does adverts interfere with the functioning of the app (if any?) | Yes/No/No adverts | Desirable not to have adverts as it may prevent the proper functioning of the app. |
|  | 8-8 | Is the app created/commissioned/ partnered by any governmental bodies/ government-affiliated companies, hospitals or universities? | Yes/No | It is desirable that the information source provided by the commission body is accredited (reliable). |
|  | 8-8-1 | If Yes, which body created/ commissioned/ partnered the app? | Free text |  |
|  | 8-8-2 | If No, is the app created/ commissioned/ partnered by an NGO/ private/ individual healthcare providers? | Yes/No/NA |  |
|  | 8-8-2-1 | Who created/ commissioned/ partnered the app? | Free text |  |
|  | 8-9 | Does the app allow recorded data of the patient to be shared on social media (e.g. Facebook/twitter?) | Yes/No | Desirable functionality for added support. |
|  | 8-10 | Does the app allow data to be exported (e.g. PDF/Excel, email, cloud, etc.)? | Yes/No | Desirable functionality for user to save data collected from app. |
|  | 8-11 | Does the app include a health professional who is accessible to the user (without users inputting any health provider/ health professional information)? | Yes/No | Desirable functionality for added support. |
|  | 8-12 | Please record your overall impressions of this app and any other observations from this app testing. | Free text | To record any significant but missing information. |
| 9. HON principles |  |  |  |  |
| Authoritative | 9-1 | Does the app indicate the qualifications of specific individuals who developed the app or contributed to the information provided? | Yes/No | The qualifications of the authors are indicated  HON principles are essential standards for information governance |

**Supplementary Table 2**: Assessment criteria (continued)

| Field | Ref | Description | Options | Justification/Evidence |
| --- | --- | --- | --- | --- |
| Complementarity | 9-2 | Is there a disclaimer stating or which implies that the information provided and/or app functions do not replace the healthcare provider's advice? | Yes/No/Advice given by healthcare provider | Information should support, not replace, the doctor-patient relationship  HON principles are essential standards for information governance |
| Privacy | 9-3 | Is there a privacy and confidentiality clause in the app? | Yes/No/NA (No personal data collected) | Respect the privacy and confidentiality of personal data submitted to the site by the visitor HON principles are essential standards for information governance |
|  | 9-3-1 | Does the app take caution regarding the collection or processing of data from children? | Yes/No/NA (not targeting children/ no data collected/ no user identifiers, e.g. age, name, user ID, photos) | How shall I treat any data gathered from children? (UK Apps developer code of conduct) With regard to apps which, because of their design or functionality, are particularly aimed at children under the age of 16 or which are particularly likely to be used by such children, you must pay attention to the age limit defining children or minors in national legislation, choose the most restrictive data processing approach in full respect of the principles of data minimization and purpose limitation, and refrain wherever possible from collecting data through children in relation to their relatives and/or friends.  Parental involvement is crucial for such apps. Therefore, you must undertake reasonable efforts to verify that consent is given or authorized by the holder of parental responsibility for the processing of health data of minors. |
|  | 9-3-2 | If yes, how does the app do so? | Free text (e.g. stated in privacy clause, pop-up before data is keyed in…) |  |
|  | 9-3-3 | If no, what patient identifiers may be collected? | Free text (e.g. name, age, date of birth, user ID, email, photographs) |  |
| Attribution | 9-4 | Are information sources in the app cited? | Yes/No/NA | Cite the source(s) of published information, date medical and health pages HON principles are essential standards for information governance |
| Justifiability | 9-5 | Are the claims relating to benefits and performance in the app store description backed up by evidence? | **Yes** (claims are backed up by evidence)/ **Inconsistent**/**No** (claims not backed up by evidence)/ **NA** (no claims) | Site must back up claims relating to benefits and performance HON principles are essential standards for information governance |
| Transparency | 9-6 | Are the developers contactable by email? (the email may be found in the app, the app store, app developer's website, etc) | Yes/No | Accessible presentation, accurate email contact HON principles are essential standards for information governance |

**Supplementary Table 2**: Assessment criteria (continued)

| Field | Ref | Description | Options | Justification/Evidence |
| --- | --- | --- | --- | --- |
| Financial disclosure | 9-7 | Does the app indicate any funding sources? | **Yes** (the app is managed by a registered commercial company) **No** (the app is managed by an individual developer without funding information) | Identify funding sources HON principles are essential standards for information governance |
| Advertising policy | 9-8 | Are advertorials distinguishable from content of the app? | Yes/No/No advertising | Clearly distinguish advertising from editorial content HON principles are essential standards for information governance |
| Assessor | 10-1 | Reviewer’s initials. (First name/Last Name) | Free text |  |
| Assessment date | 10-2 | Date the app is reviewed | dd/mm/yy |  |

*References*

NICE 2009. Depression in adults: recognition and management. Available from: www.nice.org.uk/CG91.

World Health Organization. Pharmacological treatment of mental disorders in primary health care. World Health Organization; 2009.

NICE 2004. Self-harm in over 8s: short-term management and prevention of recurrence. Available from: www.nice.org.uk/guidance/cg16 (Updated 2011)

VA/DoD clinical practice guideline for assessment and management of patients at risk for suicide. 2013

Jacobs DG, Baldessarini RJ, Conwell Y, Fawcett JA, Horton L, Meltzer H, Pfeffer CR, Simon RI. Assessment and treatment of patients with suicidal behaviors. APA Pract Guidel. 2010:1-83.
